# Economic-statistical design of synthetic *np* chart with estimated process parameter

**DOI:** 10.1371/journal.pone.0230994

**Published:** 2020-04-08

**Authors:** Ming Ha Lee, Michael B. C. Khoo, XinYing Chew, Patrick H. H. Then

**Affiliations:** 1 Faculty of Engineering, Computing and Science, Swinburne University of Technology, Kuching, Sarawak, Malaysia; 2 School of Mathematical Sciences, Universiti Sains Malaysia, Penang, Malaysia; 3 School of Computer Sciences, Universiti Sains Malaysia, Penang, Malaysia; Universita degli Studi di Genova, ITALY

## Abstract

The economic-statistical design of the synthetic *np* chart with estimated process parameter is presented in this study. The effect of process parameter estimation on the expected cost of the synthetic *np* chart is investigated with the imposed statistical constraints. The minimum number of preliminary subgroups is determined where an almost similar expected cost to the known process parameter case is desired for the given cost model parameters. However, the available number of preliminary subgroups in practice is usually limited, especially when the number of preliminary subgroups is large. Consequently, the optimal chart parameters of the synthetic *np* chart are computed by considering the practical number of preliminary subgroups in which the cost function is minimized. This leads to a lower expected cost compared to that of adopting the optimal chart parameter corresponding to the known process parameter case.

## Introduction

Control charts are generally implemented in two phases, i.e. Phase-I and Phase-II. The process parameter is estimated from an in-control reference sample in Phase-I since the process parameter is rarely known in practice. Then this estimate is used to construct the control limit for process monitoring in Phase-II. When the chart statistic plotted in Phase-II falls outside the control limit, an out-of-control signal will be triggered. In the literature, the performance of control charts with estimated process parameter has been shown to be significantly different compared to that of the known process parameter case. Therefore, considerable attention was devoted to investigate the performance of control charts when the process parameters are estimated from an in-control Phase-I dataset. For example, see [1–10].

The economic-statistical model of the synthetic *np* chart is yet to be explored by researchers in the case of process parameter estimation. Therefore, this study focuses on investigating the effect of process parameter estimation on the expected cost of the economic-statistical model of the upper-sided synthetic *np* chart for detecting increases in the process fraction non-conforming. The economic performance of the synthetic *np* chart with estimated process parameter is investigated by adopting the optimal chart parameters corresponding to the known process parameter case. In addition, the minimum number of preliminary subgroups required for the process parameter estimation is also determined such that the cost associated with the estimated process parameter case is almost similar to that of the known process parameter case. An alternative approach is also introduced in this study, where the optimal combination of chart parameters of the synthetic *np* chart is computed by minimizing the cost function, in which the number of preliminary subgroups is taken into consideration.

## Synthetic *np* chart with estimated process parameter

In this section, we derive the average run length of the upper-sided synthetic *np* chart with estimated process parameter. The Phase-I dataset comprises samples {*X*_*i*,1_, *X*_*i*,2_, *X*_*i*,3_, …, *X*_*i*,*n*_} of size *n*, for *i* = 1, 2, 3,…, *m*, where *m* is the number of preliminary subgroups. The synthetic *np* chart consists of two sub-charts: the *np* sub-chart (with upper control limit UCL) and the conforming run length sub-chart (with lower control limit *L*), where *L* is a positive integer. The process parameter is the process fraction non-conforming *p* = *τp*_0_. Note that *p* = *p*_0_ (*τ* = 1) when the process is in-control, while *p* = *p*_1_ (*τ* > 1) for detecting increases in the process fraction non-conforming when the process is out-of-control, where *τ* is the shift size.

The upper control limit of the *np* sub-chart with estimated process parameter is given as

$$\mathrm{UCL}=\left\lfloor n{\hat{p}}_{0}+k\sqrt{n{\hat{p}}_{0}(1-{\hat{p}}_{0})}\right\rfloor ,$$ (1)

where ⌊⋅⌋ denotes the rounded down integer, *k* is the coefficient of the UCL and ${\hat{p}}_{0}=\frac{x}{mn}$ is an estimator of process parameter *p*_0_, for *x* ∈ {0, 1, 2, …, *mn*– 1, *mn*}. Let $\hat{\theta }$ be the probability that a chart statistic (i.e. number *X*_*i*_ of non-conforming units in the sample) is plotted above UCL, then

$$\begin{array}{l} \hat{\theta }=1-\mathrm{Pr}(X_{i}\leq \mathrm{UCL})\\ \;=1-\mathrm{Pr}\left(X_{i}\leq \left\lfloor n{\hat{p}}_{0}+k\sqrt{n{\hat{p}}_{0}(1-{\hat{p}}_{0})}\right\rfloor \right)\\ \;=1-\mathrm{Pr}\left(\left\lfloor n{\hat{p}}_{0}+k\sqrt{n{\hat{p}}_{0}(1-{\hat{p}}_{0})}\right\rfloor \right)\\ \;=1-F(\left\lfloor \frac{x}{m}+k\sqrt{\frac{x}{m}\left(1-\frac{x}{mn}\right)}\right\rfloor |n,p), \end{array}$$

where *F*(⋅|*n*,*p*) is the cumulative distribution function of a binomial random variable with parameters (*n*, *p*).

Let $A\hat{R}L=\frac{1}{\hat{\theta }(1-{\left(1-\hat{\theta }\right)}^{L})}$ be the conditional average run length of the synthetic *np* chart with estimated process parameter and let *f*(*x*|*mn*,*p*_0_) be the probability density function of a binomial random variable with parameters (*mn*, *p*_0_). Then the unconditional average run length of the synthetic *np* chart with estimated process parameter is calculated as

$$\mathrm{ARL}=\sum_{x=x_{\min}}^{x_{\max}}A\hat{R}L\times f(x|mn,p_{0}),$$ (2)

where $x_{\min}=\max\left(0,\left\lfloor mnp_{0}-10\sqrt{mnp_{0}(1-p_{0})}\right\rfloor \right)$ and $x_{\max}={\left\lceil mnp_{0}-10\sqrt{mnp_{0}(1-p_{0})}\right\rceil }^{}$. As in [11–13], the same formulae of *x*_min_ and *x*_max_ are used to compute the ARL for the *np* type chart with estimated process parameter. Note that the unconditional ARL in Eq (2) is derived as a summation of the product of the conditional ARL (i.e. $A\hat{R}L$) and the in-control probability mass function of the binomial random variable (i.e. *f*(*x*|*mn*,*p*_0_)) since this probability is related to Phase-I with the process parameter of *p*_0_ [12]. Here, ARL = ARL_0_ (*τ* = 1) when the process is in-control, while ARL = ARL_1_ (*τ* > 1) when the process is out-of-control. Note that when the process parameter is known, the average run length of the synthetic *np* chart is

$$\mathrm{ARL}=\frac{1}{\theta (1-{\left(1-\theta \right)}^{L})},$$ (3)

where *θ* = 1−*F*(UCL|*n*,*p*). Here, $\mathrm{UCL}=\left\lfloor np_{0}+k\sqrt{np_{0}(1-p_{0})}\right\rfloor$.

### Economic-statistical design of the synthetic *np* chart

Numerous research works on the economic and economic-statistical designs of control charts were using the cost model developed by Lorenzen and Vance [14]. For example, see [15–22]. The Lorenzen and Vance’s cost model is a general model applicable to various types of control charts regardless of the statistic used and the calculation of the statistical properties requires the ARL criterion. In this study, the cost function in Lorenzen and Vance [14] is used for the economic-statistical design of the synthetic *np* chart with estimated process parameter and the expected cost per unit time of the cost model is calculated as

$$\mathrm{cost}=\frac{C_{0}/\lambda +C_{1}[-\phi +nE+{\mathrm{ARL}}_{1}h+r_{1}T_{1}+r_{2}T_{2}]+sY/{\mathrm{ARL}}_{0}+W}{1/\lambda +(1-r_{1})sT_{0}/{\mathrm{ARL}}_{0}-\phi +nE+{\mathrm{ARL}}_{1}h+T_{1}+T_{2}}$$

$$+\frac{\frac{a+bn}{h}(1/\lambda -\phi +nE+{\mathrm{ARL}}_{1}h+r_{1}T_{1}+r_{2}T_{2})}{1/\lambda +(1-r_{1})sT_{0}/{\mathrm{ARL}}_{0}-\phi +nE+{\mathrm{ARL}}_{1}h+T_{1}+T_{2}},$$ (4)

where *C*_0_ is the cost of producing non-conformities when the process is in-control; *λ* is the process failure rate; *C*_1_ is the cost of producing non-conformities when the process is out-of-control; $\phi =\frac{1}{\lambda }-\frac{h}{e^{\lambda h}-1}$ is the expected time of occurrence of an assignable causes; *E* is the time to sample and chart one item; *h* is the sampling interval; *r*_1_ = 1 if the production continues during the search for an assignable cause, while *r*_1_ = 0 if the production stops during the search for an assignable cause; *r*_2_ = 1 if the production continues during repair, while *r*_2_ = 0 if the production stops during repair; *T*_1_ is the expected time to find an assignable cause; *T*_2_ is the expected time to repair the process; $s=\frac{1}{e^{\lambda h}-1}$ is the expected number of samples taken when the process is in-control; *Y* is the cost per false alarm; *W* is the cost to locate an assignable cause and repair the process; *a* is the fixed cost per sample; *b* is the variable cost per unit sampled; and *T*_0_ is the expected time spent searching for a false alarm.

The following statistical constraints are imposed to achieve the required statistical performance of the synthetic *np* chart:

$${\mathrm{ARL}}_{0}\geq {\mathrm{ARL}}_{L}\;\mathrm{and}\;{\mathrm{ARL}}_{1}\leq {\mathrm{ARL}}_{U},$$ (5)

where ARL_L_ is the desired lower limit of the in-control statistical constraint and ARL_U_ is the desired upper limit of the out-of-control statistical constraint. The optimal chart parameters (*k*, *L*, *n*, *h*) of the synthetic *np* chart are obtained using Evolver (add-in Microsoft Excel solver), in which the expected cost in Eq (4) is minimized with the imposed statistical constraints in Eq (5), subject to *k* > 0.01, *L* ≥ 1, *n* ≥ 1 and 0.01 < *h* < 8, where *L* and *n* are positive integers.

## Effect of the process parameter estimation on the expected cost

In this study, the input parameters of the cost function for the economic-statistical design of the synthetic *np* chart are the cost model parameters as follows: *λ* ∈ {0.01, 0.02, 0.03}, *C*_0_ ∈ {57.12, 114.24, 171.36}, *C*_1_ ∈ {474.6, 949.2, 1423.8}, *E* ∈ {0.04167, 0.08333, 0.125}, *T*_0_ ∈ {0.04167, 0.08333, 0.125}, *T*_1_ ∈ {0.04167, 0.08333, 0.125}, *T*_2_ ∈ {0.375, 0.75, 1.125}, *Y* ∈ {488.7, 977.4, 1466.1}, *W* ∈ {488.7, 977.4, 1466.1}, *a* ∈ {0, 5, 10} and *b* ∈ {2.11, 4.22, 6.33}. It is assumed that *r*_1_ = 1 (i.e. the production continues during the search for an assignable cause) and *r*_2_ = 0 (i.e. the production stops during repair) with *p*_0_ = 0.02 and *τ* ∈ {1.4, 1.6, 1.8, 2, 2.2, 2.4, 2.6}, where ARL_L_ = 200 and ARL_U_ = 5. Table 1 lists the values of these parameters for 29 cases with the bolded value in each row indicating the parameter that is varied for each case. Table 2 lists the optimal chart parameters of the synthetic *np* chart with the corresponding values of ARL_0_, ARL_1_ and expected cost under the assumption of the known process parameter. The optimal chart parameters in Table 2 are used to compute the expected cost of the synthetic *np* chart with different values of *m* ∈ {10, 20, 50, 100} for the estimated process parameter case and *m* = ∞ for the known process parameter case, where these expected costs are provided in Table 3. In comparison to the expected cost of the synthetic *np* chart with known process parameter, the difference in the expected cost (difference in cost in Table 3) of the synthetic *np* chart with estimated process parameter computed using the optimal chart parameters of the known process parameter case is calculated as cost_*m*_−cost_∞_, where cost_*m*_ is the expected cost of the estimated process parameter case and cost_∞_ is the expected cost of the known process parameter case. Note that a positive value indicates that the expected cost of the estimated process parameter case is higher than that of the known process parameter case, while a negative value indicates that the expected cost of the estimated process parameter case is lower than that of the known process parameter case.

**Table 1:** Input parameters.

Case	*τ*	*λ*	*C*_0_	*C*_1_	*E*	*T*_0_	*T*_1_	*T*_2_	*Y*	*W*	*a*	*b*
1	**1.4**	0.02	114.24	949.2	0.08333	0.08333	0.08333	0.75	977.4	977.4	0	4.22
2	**1.6**	0.02	114.24	949.2	0.08333	0.08333	0.08333	0.75	977.4	977.4	0	4.22
3	**1.8**	0.02	114.24	949.2	0.08333	0.08333	0.08333	0.75	977.4	977.4	0	4.22
4	**2**	0.02	114.24	949.2	0.08333	0.08333	0.08333	0.75	977.4	977.4	0	4.22
5	**2.2**	0.02	114.24	949.2	0.08333	0.08333	0.08333	0.75	977.4	977.4	0	4.22
6	**2.4**	0.02	114.24	949.2	0.08333	0.08333	0.08333	0.75	977.4	977.4	0	4.22
7	**2.6**	0.02	114.24	949.2	0.08333	0.08333	0.08333	0.75	977.4	977.4	0	4.22
8	2	**0.01**	114.24	949.2	0.08333	0.08333	0.08333	0.75	977.4	977.4	0	4.22
9	2	**0.03**	114.24	949.2	0.08333	0.08333	0.08333	0.75	977.4	977.4	0	4.22
10	2	0.02	**57.12**	949.2	0.08333	0.08333	0.08333	0.75	977.4	977.4	0	4.22
11	2	0.02	**171.36**	949.2	0.08333	0.08333	0.08333	0.75	977.4	977.4	0	4.22
12	2	0.02	114.24	**474.6**	0.08333	0.08333	0.08333	0.75	977.4	977.4	0	4.22
13	2	0.02	114.24	**1423.8**	0.08333	0.08333	0.08333	0.75	977.4	977.4	0	4.22
14	2	0.02	114.24	949.2	**0.04167**	0.08333	0.08333	0.75	977.4	977.4	0	4.22
15	2	0.02	114.24	949.2	**0.12500**	0.08333	0.08333	0.75	977.4	977.4	0	4.22
16	2	0.02	114.24	949.2	0.08333	**0.04167**	0.08333	0.75	977.4	977.4	0	4.22
17	2	0.02	114.24	949.2	0.08333	**0.12500**	0.08333	0.75	977.4	977.4	0	4.22
18	2	0.02	114.24	949.2	0.08333	0.08333	**0.04167**	0.75	977.4	977.4	0	4.22
19	2	0.02	114.24	949.2	0.08333	0.08333	**0.12500**	0.75	977.4	977.4	0	4.22
20	2	0.02	114.24	949.2	0.08333	0.08333	0.08333	**0.375**	977.4	977.4	0	4.22
21	2	0.02	114.24	949.2	0.08333	0.08333	0.08333	**1.125**	977.4	977.4	0	4.22
22	2	0.02	114.24	949.2	0.08333	0.08333	0.08333	0.75	**488.7**	977.4	0	4.22
23	2	0.02	114.24	949.2	0.08333	0.08333	0.08333	0.75	**1466.1**	977.4	0	4.22
24	2	0.02	114.24	949.2	0.08333	0.08333	0.08333	0.75	977.4	**488.7**	0	4.22
25	2	0.02	114.24	949.2	0.08333	0.08333	0.08333	0.75	977.4	**1466.1**	0	4.22
26	2	0.02	114.24	949.2	0.08333	0.08333	0.08333	0.75	977.4	977.4	**5**	4.22
27	2	0.02	114.24	949.2	0.08333	0.08333	0.08333	0.75	977.4	977.4	**10**	4.22
28	2	0.02	114.24	949.2	0.08333	0.08333	0.08333	0.75	977.4	977.4	0	**2.110**
29	2	0.02	114.24	949.2	0.08333	0.08333	0.08333	0.75	977.4	977.4	0	**6.330**

**Table 2:** Optimal chart parameters and the corresponding ARL_0_, ARL_1_ and expected cost of the economic-statistical design of the synthetic *np* chart with known process parameter.

Case	*k*	*L*	*n*	*h*	ARL_0_	ARL_1_	cost
1	2.1447	11	492	8.00	200.28	4.80	879.06
2	2.1533	9	241	8.00	204.70	4.42	670.39
3	2.1939	9	142	5.02	200.42	4.28	560.33
4	2.2560	9	82	3.13	202.91	4.78	475.96
5	2.2717	8	56	2.38	205.44	4.90	420.15
6	2.2717	8	56	2.67	205.44	3.82	393.76
7	2.3486	8	32	1.66	205.94	4.94	350.36
8	2.2560	9	82	3.88	202.91	4.78	369.09
9	2.2857	10	81	2.83	201.95	4.86	551.77
10	2.2857	10	81	2.96	201.95	4.86	435.60
11	2.2560	9	82	3.28	202.91	4.78	515.96
12	2.2560	9	82	5.54	202.91	4.78	323.31
13	2.2857	10	81	2.34	201.95	4.86	602.47
14	2.2560	9	82	2.95	202.91	4.78	446.14
15	2.2857	10	81	3.27	201.95	4.86	502.17
16	2.2560	9	82	3.13	202.91	4.78	475.96
17	2.2560	9	82	3.13	202.91	4.78	475.96
18	2.2857	10	81	3.08	201.95	4.86	475.61
19	2.2560	9	82	3.14	202.91	4.78	476.30
20	2.2560	9	82	3.14	202.91	4.78	478.48
21	2.2560	9	82	3.13	202.91	4.78	473.46
22	2.2560	9	82	3.12	202.91	4.78	475.43
23	2.2560	9	82	3.15	202.91	4.78	476.48
24	2.2857	10	81	3.06	201.95	4.86	469.07
25	2.2560	9	82	3.16	202.91	4.78	482.82
26	2.2560	9	82	3.16	202.91	4.78	477.53
27	2.2560	9	82	3.19	202.91	4.78	479.08
28	2.2857	10	81	2.06	201.95	4.86	409.77
29	2.2266	8	83	4.09	206.53	4.73	524.54

**Table 3:** Expected cost of the synthetic *np* chart with estimated process parameter corresponding to the optimal chart parameters of the known process parameter case.

Case	*m* = 10		*m* = 20		*m* = 50		*m* = 100		*m* = ∞
	Cost	Difference in cost	Cost	Difference in cost	Cost	Difference in cost	Cost	Difference in cost	Cost
1	912.91	33.85	893.81	14.75	885.44	6.38	882.43	3.37	879.06
2	721.77	51.38	691.78	21.39	679.17	8.78	674.44	4.05	670.39
3	614.04	53.71	587.40	27.07	570.30	9.97	564.13	3.80	560.33
4	540.18	64.22	503.49	27.53	487.52	11.56	478.69	2.73	475.96
5	491.01	70.86	450.56	30.41	430.14	9.99	421.90	1.75	420.15
6	440.44	46.68	413.83	20.07	400.18	6.42	394.85	1.09	393.76
7	422.13	71.77	377.39	27.03	359.24	8.88	351.36	1.00	350.36
8	422.93	53.84	391.70	22.61	378.49	9.40	371.30	2.21	369.09
9	628.35	76.58	584.75	32.98	564.01	12.24	554.64	2.87	551.77
10	511.37	75.77	467.60	32.00	447.34	11.74	438.33	2.73	435.60
11	577.18	61.22	542.27	26.31	527.03	11.07	518.58	2.62	515.96
12	357.60	34.29	338.53	15.22	329.81	6.50	324.87	1.56	323.31
13	700.37	97.90	643.27	40.80	617.34	14.87	605.92	3.45	602.47
14	514.35	68.21	475.36	29.22	458.40	12.26	449.03	2.89	446.14
15	570.64	68.47	531.18	29.01	512.84	10.67	504.66	2.49	502.17
16	540.18	64.22	503.49	27.53	487.52	11.56	478.69	2.73	475.96
17	540.18	64.22	503.49	27.53	487.52	11.56	478.69	2.73	475.96
18	548.11	72.50	506.30	30.69	486.89	11.28	478.24	2.63	475.61
19	540.48	64.18	503.82	27.52	487.85	11.55	479.03	2.73	476.30
20	542.77	64.29	506.05	27.57	490.06	11.58	481.22	2.74	478.48
21	537.57	64.11	500.93	27.47	484.99	11.53	476.19	2.73	473.46
22	540.03	64.60	503.30	27.87	487.22	11.79	478.26	2.83	475.43
23	540.34	63.86	503.68	27.20	487.82	11.34	479.13	2.65	476.48
24	542.09	73.02	499.97	30.90	480.43	11.36	471.72	2.65	469.07
25	546.54	63.72	510.16	27.34	494.31	11.49	485.54	2.72	482.82
26	542.07	64.54	505.22	27.69	489.16	11.63	480.28	2.75	477.53
27	543.94	64.86	506.93	27.85	490.79	11.71	481.86	2.78	479.08
28	465.99	56.22	432.73	22.96	417.93	8.16	411.60	1.83	409.77
29	593.23	68.69	561.61	37.07	538.55	14.01	527.95	3.41	524.54

In Table 3, it is noticeable that the expected cost of the synthetic *np* charts with estimated process parameter (*m* = 10, 20, 50, 100) is higher compared to that of the corresponding chart with known process parameter (*m* = ∞) since the differences in the expected costs are all positive values. For example, the optimal chart parameters in Case 4 are (*k*, *L*, *n*, *h*) = (2.2560, 9, 82, 3.13) when the process parameter is known and the expected cost is computed as $475.96. By adopting these chart parameters, that are (*k*, *L*, *n*, *h*) = (2.2560, 9, 82, 3.13), the expected costs per hour are $540.18, $503.49, $487.52 and $478.69 for the estimated process parameter cases with number of subgroups *m* = 10, 20, 50 and 100, respectively. Therefore, the expected costs have increased by $64.22, $27.53, $11.56 and $2.73 for *m* = 10, 20, 50 and 100, respectively when the optimal chart parameters corresponding to the known process parameter case are used to compute the expected cost of estimated process parameter case. This shows that the increase in the expected cost is larger when the value of *m* is smaller, indicating that the expected cost of the estimated process parameter case deviates significantly from that of the known process parameter case, especially when the value of *m* is small.

## Effect of the number of preliminary subgroups on the expected cost

The results in the previous section show that when *m* decreases, the difference between the expected cost for known and estimated process parameter cases increases. Therefore, it is inappropriate to calculate the expected cost of the estimated process parameter case by adopting the optimal chart parameters associated with the known process parameter case. For this reason, the minimum number of preliminary subgroups in Phase-I (i.e. minimum value of *m*) is determined in this section, such that the performance of the *np* chart with estimated process parameter is approximately similar to that of the corresponding chart with known process parameter. Consequently, the minimum value of *m* is determined in order to obtain a nearly similar expected cost as the known process parameter case, in which the percentage of difference in the expected cost between the known and estimated process parameter cases is less than 0.01%, where the formulation is given as follows:

$$\Delta =\frac{{\mathrm{cost}}_{m}-{\mathrm{cost}}_{\infty }}{{\mathrm{cost}}_{\infty }}\times 100<0.01,$$ (6)

where cost_*m*_ denotes the expected cost associated with the estimated process parameter case and cost_∞_ denotes the expected cost associated with the known process parameter case.

Table 4 provides the minimum value of *m* of the estimated process parameter based synthetic *np* chart, matching approximately a similar expected cost value to the corresponding chart with known process parameter, where the chart parameters in Table 2 are considered. In Table 4, it can be seen that the minimum values of *m* satisfying Eq (6) for all the given cases are larger than 250, except for Cases 5–7 when the shift size is large (*τ* ≥ 2.2).

**Table 4:** Minimum values of *m* satisfying Δ < 0.01.

Case	Minimum *m* value
1	527
2	411
3	321
4	265
5	229
6	218
7	191
8	266
9	260
10	274
11	262
12	256
13	271
14	273
15	259
16	265
17	265
18	271
19	265
20	265
21	265
22	266
23	264
24	266
25	264
26	265
27	265
28	257
29	267

## Optimal design of the cost model with estimated process parameter

In practice, practitioners are usually interested to use a small number of preliminary subgroups to estimate the process parameter in Phase-I since using a large number of Phase-I samples can be practically infeasible. To address this issue, an alternative approach is proposed in this study, where a model for the economic-statistical design is developed by computing the optimal chart parameters which take the number of preliminary subgroups into account when designing the synthetic *np* chart with estimated process parameter. For this alternative approach, the optimal chart parameters of the synthetic *np* chart with estimated process parameter are computed by minimizing the expected cost. The optimization procedure of the economic-statistical design of the synthetic *np* chart with estimated process parameter is illustrated as follows:

Step 1. Specify the values of *m* and *τ*.
Step 2. Determine the values of the cost model parameters, which are *C*_0_, *C*_1_, *E*, *T*_0_, *T*_1_, *T*_2_, *Y*, *W*, *a*, *b*, *r*_1_ and *r*_2_.
Step 3. Determine the statistical constraints in Eq (5).
Step 4. Calculate UCL using Eq (1).
Step 5. Calculate ARL_0_ (when *τ* = 1) and ARL_1_ (when *τ* > 1) using Eq (2).
Step 6. Minimize the cost function in Eq (4) to obtain the lowest expected cost.
Step 7. The chart parameters (*k*, *L*, *n*, *h*) associated with the lowest expected cost are the optimal chart parameters for the synthetic *np* chart with estimated process parameter. These optimal chart parameters can be used to compute the control limit of the synthetic *np* chart for Phase-II process monitoring.

By employing the aforementioned procedure, Table 5 summarizes the expected cost of the synthetic *np* chart for *m* = 10, 20, 50 and 100 associated with the optimal chart parameters of the estimated process parameter case, instead of adopting the optimal chart parameters corresponding to the known process parameter case. In Table 5, it can be seen that with the implementation of the new design model, the synthetic *np* chart has a lower expected cost compared with adopting the chart parameters corresponding to the known process parameter case. For example, by referring to Case 8, the expected cost computed using the optimal chart parameters associated with the known process parameter case are $422.93, $391.70, $378.49 and $371.30 for *m* = 10, 20, 50 and 100, respectively (see Table 3); while the expected cost obtained by adopting the optimal chart parameter corresponding to the estimated process parameter case are $313.24, $338.40, $346.94 and $346.68 for *m* = 10, 20, 50 and 100, respectively (see Table 5). Therefore, the optimal chart parameters computed based on the new design model allow a reduction in the expected cost compared with using the chart parameters corresponding to the known process parameter case.

**Table 5:** Expected cost of the synthetic *np* chart with estimated process parameter corresponding to the optimal chart parameters of the estimated process parameter case.

Case	*m* = 10	*m* = 20	*m* = 50	*m* = 100	*m* = ∞
1	773.96	813.29	837.57	844.24	879.06
2	586.38	624.49	640.42	642.41	670.39
3	470.98	509.56	530.66	538.62	560.33
4	400.33	434.60	447.35	445.80	475.96
5	344.49	378.75	385.28	388.72	420.15
6	309.51	339.68	362.67	359.15	393.76
7	286.37	320.43	322.06	330.49	350.36
8	313.24	338.40	346.94	346.68	369.09
9	465.10	504.76	517.51	517.60	551.77
10	355.84	391.92	403.64	403.73	435.60
11	444.55	476.99	487.48	487.56	515.96
12	285.93	303.37	308.85	308.84	323.31
13	493.38	542.41	558.32	558.43	602.47
14	379.57	410.24	419.88	419.79	446.14
15	419.62	457.09	469.15	469.23	502.17
16	400.33	434.73	445.71	445.80	475.96
17	400.33	434.73	445.71	445.80	475.96
18	399.91	434.21	445.34	445.42	475.61
19	400.74	434.98	446.09	446.17	476.30
20	402.67	437.04	448.59	448.26	478.48
21	402.50	432.18	444.08	443.36	473.46
22	399.46	433.92	445.93	445.15	475.43
23	401.19	435.27	446.36	446.44	476.48
24	392.73	427.31	438.54	438.62	469.07
25	407.92	441.87	452.88	452.97	482.82
26	402.63	436.57	447.57	447.66	477.53
27	404.89	438.51	449.41	449.50	479.08
28	344.32	373.40	383.10	383.15	409.77
29	441.75	479.48	491.52	491.62	524.54

## Conclusions

In this study, a model for the economic-statistical design of the synthetic *np* chart with estimated process parameter is presented. The results show that the expected cost is affected by the parameter estimation, where the expected cost of the synthetic *np* chart with estimated process parameter is significantly different compared with the corresponding known process parameter chart, especially when the number of preliminary subgroups (i.e. value of *m*) is small. The expected cost for the estimated process parameter case (*m* < ∞) is higher than the expected cost corresponding to the case of known process parameter case (*m* = ∞). It can also be noticed that the expected cost decreases with a larger value of *m*.

This study suggests the minimum number of preliminary subgroups for the synthetic *np* chart with estimated process parameter to have an almost similar expected cost to the known process parameter case. The results show that the minimum number of preliminary subgroups is large (i.e. more than 250) in most cases. This shows that a larger number of preliminary subgroups is required in order to have the expected cost that is almost similar to that of the *np* chart with known process parameter. An alternative approach in designing the synthetic *np* chart with estimated process parameter is also introduced by computing the optimal chart parameters, subject to the imposed statistical constraints so that the expected cost is minimized.
